# Adoption of C-reactive protein point-of-care tests for the management of acute childhood infections in primary care in the Netherlands and England: a comparative health systems analysis

**DOI:** 10.1186/s12913-023-09065-8

**Published:** 2023-02-23

**Authors:** Juan Emmanuel Dewez, Ruud G. Nijman, Elizabeth J. A. Fitchett, Rebecca Lynch, Ronald de Groot, Michiel van der Flier, Ria Philipsen, Harriet Vreugdenhil, Stefanie Ettelt, Shunmay Yeung

**Affiliations:** 1 Clinical Research Department, London School of Hygiene & Tropical Medicine, London, UK; 2 Department of Infectious Diseases, Section of Paediatric Infectious Diseases, Imperial College London, London, UK; 3 Wellcome Centre for Cultures and Environments of Health, University of Exeter, Exeter, UK; 4 Section of Paediatric Infectious Diseases, Laboratory of Medical Immunology, Radboud Centre for Infectious Diseases, Radboud Institute for Molecular Life Sciences, Radboud UMC, Nijmegen, The Netherlands; 5 Paediatric Infectious diseases and Immunology, Amalia Children’s Hospital, Radboudumc, Nijmegen, The Netherlands; 6 Paediatric Infectious Diseases and Immunology, Wilhelmina Children’s Hospital, University Medical Center Utrecht, Utrecht, The Netherlands; 7 Utrecht General Practice Training Institute, University Medical Center Utrecht, Utrecht, The Netherlands; 8 Department of Health Services Research and Policy, London School of Hygiene & Tropical Medicine, London, UK; 9 Prognos AG, Basel, Switzerland; 10 Department of Paediatrics, St Mary’s Imperial College Hospital NHS Trust, London, UK

**Keywords:** Comparative health systems analysis, NASSS framework, C-reactive protein, Point-of-care tests, The Netherlands, England, Acute childhood infections, Primary care

## Abstract

**Background:**

The use of point of care (POC) tests varies across Europe, but research into what drives this variability is lacking. Focusing on CRP POC tests, we aimed to understand what factors contribute to high versus low adoption of the tests, and also to explore whether they are used in children.

**Methods:**

We used a comparative qualitative case study approach to explore the implementation of CRP POC tests in the Netherlands and England. These countries were selected because although they have similar primary healthcare systems, the availability of CRP POC tests in General Practices is very different, being very high in the former and rare in the latter. The study design and analysis were informed by the non-adoption, abandonment, spread, scale-up and sustainability (NASSS) framework. Data were collected through a review of documents and interviews with stakeholders. Documents were identified through a scoping literature review, search of websites, and stakeholder recommendation. Stakeholders were selected purposively initially, and then by snowballing. Data were analysed thematically.

**Results:**

Sixty-five documents were reviewed and 21 interviews were conducted. The difference in the availability of CRP POC tests is mainly because of differences at the wider national context level. In the two countries, early adopters of the tests advocated for their implementation through the generation of robust evidence and by engaging with all relevant stakeholders. This led to the inclusion of CRP POC tests in clinical guidelines in both countries. In the Netherlands, this mandated their reimbursement in accordance with Dutch regulations. Moreover, the prevailing better integration of health services enabled operational support from laboratories to GP practices. In England, the funding constraints of the National Health Service and the prioritization of alternative and less expensive antimicrobial stewardship interventions prevented the development of a reimbursement scheme. In addition, the lack of integration between health services limits the operational support to GP practices. In both countries, the availability of CRP POC tests for the management of children is a by-product of the test being available for adults. The tests are less used in children mainly because of concerns regarding their accuracy in this age-group.

**Conclusions:**

The engagement of early adopters combined with a more favourable and receptive macro level environment, including the role of clinical guidelines and their developers in determining which interventions are reimbursed and the operational support from laboratories to GP practices, led to the greater adoption of the tests in the Netherlands. In both countries, CRP POC tests, when available, are less used less in children. Organisations considering introducing POC tests into primary care settings need to consider how their implementation fits into the wider health system context to ensure achievable plans.

**Supplementary Information:**

The online version contains supplementary material available at 10.1186/s12913-023-09065-8.

## Background

Fever is a common reason for paediatric consultations in primary care [1]. Most febrile children have self-limiting infections [2, 3], but differentiating the few febrile children with severe bacterial infections from those with minor illness is difficult because the clinical features of infection in children are often non-specific. The resulting diagnostic uncertainty combined with avoidance of risk lead to the over-prescription of antibiotics [4], which may contribute to antimicrobial resistance [5].

Point-of-care (POC) tests have been widely advocated to reduce antibiotic resistance [5]. They can be easily performed in the consultation room, provide rapid results, and may optimise antibiotics use and patient care.

Few POC tests are used in the clinical management of acute fever in children, and their performance and impact seem to vary [6]. These include urine dipsticks to diagnose urinary tract infections, rapid throat tests to identify Group A Streptococcal infections, and C-reactive protein (CRP) POC tests.

CRP is a non-specific marker of acute inflammation used to indicated the severity of infections [7]. It is one of the most widely used and studied biomarkers in the management of infections [8].

The clinical accuracy and effectiveness of using CRP POC tests in primary care have been studied extensively, mainly in the management of adults. Recent systematic reviews have concluded that the use of the tests can help to reduce antibiotic prescription in adults with respiratory tract infections. With regards the use of the tests in children, it also reduces antibitoc prescription, but only if guidance is provided [9, 10]. However, the cost-effectiveness of using CRP POC tests and the broader factors that influence their implementation in routine practice, such as clinicians’ attitudes, funding, quality assurance, impact on workload, or regulation [9, 11], have received less attention [12]. The availability of CRP POC tests in primary care varies across Europe with higher availability in Scandinavian countries, Switzerland, and the Netherlands compared to England or other countries [13, 14]. Moreover, whether CRP POC tests are used in the management of acute childhood infections is unclear.

Understanding the mechanisms that influence the availability and use of CRP POC tests is important to inform the implementation of current and future POC tests for the management of acute childhood infections. The aims of this study were to generate an in-depth understanding of the factors that contributed to a high versus a low availability of CRP POC tests in two countries with similar primary healthcare systems; and to explore whether the tests are used in children.

## Methods

**Fig. 1:**
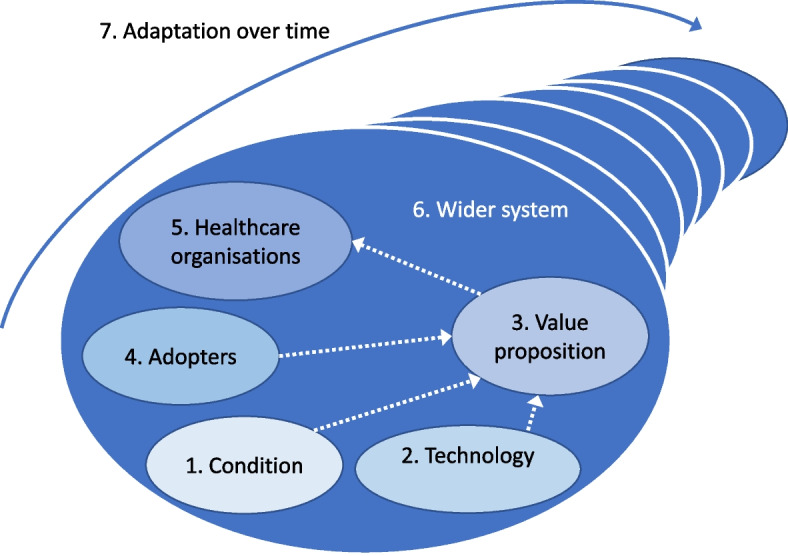
The non-adoption, abandonment, spread, scale-up and sustainability of healthcare technologies (NASSS) framework (adapted from Greenhalgh et al.) [15]

A comparative qualitative analysis based on two country case studies of the implementation of CRP POC tests was conducted. This approach was chosen as it allows for an in-depth understanding of a multifaceted phenomenon such as the introduction of diagnostics, which involves multiple actors and processes within a wider national context. The design of the study was informed by the non-adoption, abandonment, spread, scale-up and sustainability of healthcare technologies (NASSS) framework [15]. The NASSS framework was developed to identify factors that contribute to the adoption of innovations in healthcare services by assessing the complexity of seven domains: (1) the condition or illness; (2) the technology; (3) the value of the innovation for developers and users; (4) the adopters and whether the innovation implied a change in their identity and practices; (5) the organisations where the innovation is implemented, whether they are ready for this innovation, how the innovation changes the organisations’ routines, and the work needed to adopt, fund, and normalise the innovation; (6) the wider context including the policy and regulatory contexts, the role of professional bodies and interorganisational networking; and (7) the adaptation of the innovation, its use, and the organisations over time (Fig. 1).

The two countries that were purposively selected for the comparison were the Netherlands and England. The criterion used to make this selection was to allow for a “more similar” type of comparison [16], i.e., countries where there is a substantial difference in the outcome of interest (high availability of CRP POC tests in primary care in the Netherlands and very low in England) [13, 17] but where the context are similar with regards the organisation of primary care services and in overall health expenditure. In both countries general practitioners (GPs) provide primary care for children, are the gatekeepers of health services, and health expenditure is similar at around 10% of GDP [18]. An additional criterion was feasibility in terms of working within an established collaboration.

Data were collected through an iterative process combining document analysis and interviews with stakeholders. The initial document analysis sought to explore the wider health system contexts and to inform the identification of relevant stakeholders and the development of topic guides (supplementary material 1). This was followed by interviews of stakeholders and additional document analyses. The iterative combination of these two methods allowed the triangulation of data for two purposes: (1) to cross-validate findings and (2) to extend the understanding of findings.

Documents were included if they pertained to the adoption of CRP POC tests in the two countries and were published after 2000. Documents included publications in medical journals, clinical guidelines, information for patients, information for implementors of diagnostic tests, reports from healthcare organisations, minutes of meetings, and proceedings of conferences. Documents were identified through a multi-pronged approach: a scoping review of the literature; an extensive search of the websites of relevant healthcare organisations; interviewee recommendations; and through attendance at relevant meetings (see supplementary material 2 for additional details).

Stakeholders were selected based on their expert knowledge of at least one domains of the NASSS framework pertaining to the adoption of CRP POC tests in primary care in their country. We also ensured that we had at least one representative of the three levels of health systems: micro (stakeholders who used/could use CRP POC tests), meso (stakeholders directly involved in the implementation of diagnostics in GP practices) and macro (stakeholders involved in the wider national context). Based on the inclusion criteria, potential interviewees were identified through personal contacts; searching authors of relevant reports; and in the UK by attending relevant conferences. Initial interviewees were sampled purposively followed by snowball sampling to identify additional stakeholders that could provide insights on domains of the NASSS framework that were not covered in initial interviews.

In the Netherlands, the interviewees were based in Nijmegen where members of the research team worked, and in Eindhoven, Leusden and Utrecht. In England, interviewees worked in Hertfordshire, Herefordshire, Southampton, and London. Potential participants were contacted by email or telephone to ascertain their interest in being interviewed. Those who agreed, were followed-up by JED who provided a participant information sheet, obtained written informed consent, and arranged the interview date.

JED conducted all the interviews, with SY participating in one interview in the Netherlands. The interviewers did not know participants beforehand. Face-to-face audio recorded interviews took place at the respondents’ workplace between March 2019 and February 2020, and by videoconference between March 2020 and August 2021 because of the restriction due to the COVID-19 pandemic. Only the interviewers and the participants were present during the interview. All interview records were transcribed verbatim by a research assistant or JED. Field notes were taken after each interview. One transcript was returned to a participant who requested this; no corrections were made. Two participants were recontacted to clarify the information provided in the interviews. No repeat interviews were conducted.

The documents and interview transcripts were analysed thematically. The analysis was deductive based on the seven domains of the NASSS framework. JED extracted data from the interview transcripts and documents and collated them per NASSS domain using matrices in Excel, including alternative views, when available. EF independently assessed whether each extract was assigned to the most relevant NASSS domains. Discrepancies were resolved through discussion and consensus between JED and EF. Data from the two countries were analysed separately. A summary of each domain was produced and the summaries of the two countries were then compared descriptively to highlight similarities and differences for each domain. All authors verified the consistency of each domain summary. Data saturation was considered reached when all domains of the NASSS framework were covered and each domain was clearly understood. Participants did not provide feedback on the findings.

## Results

Sixty-five documents including research publications, clinical guidelines, reimbursement decisions, health systems reviews, and policies were included in the analysis (Table 1). A total of 21 stakeholders were interviewed, including GPs, POCT implementors (i.e., the head of a laboratory implementing POC tests in primary care, and a nurse in charge of implementing a pilot study with CRP POC tests) and representatives of a Clinical Commissioning Group, a health insurance company, NHS improvement (a professional body supporting quality improvement in the English National Health Service), clinical guideline development bodies, and the in-vitro diagnostics industry (Table 2). All the included GPs from the Netherlands used CRP POC tests because despite our efforts we were unable to identify GPs who did not; in England four of the six GPs had used the test as part of pilot studies. Three GP practices did not reply to the invitation: one in the Netherlands and two in England. Four successive industry representatives did not reply to the invitation in England. Interviews lasted 32–73 minutes.

**Table 1:** Documents included in the analysis

Author and year	Title	Type of document	NASSS domains
Hay, 2005 [1]	The prevalence of symptoms and consultations in pre-school children in the Avon Longitudinal Study of Parents and Children.	Observational study aiming to describe symptom and consultation prevalence in pre-school children.	Domain 1
De Bont, 2015 [19]	Workload and management of childhood fever at general practice out-of-hours care.	Observational study aiming to describe the work of out of hours GPs.	Domain 1 and 5
Veldhoen, 2009 [20]	Changes in infectious disease mortality among children in the Netherlands.	Observational study aiming to examine the changes in mortality due to infectious diseases in childhood over recent decades in the Netherlands.	Domain 1
Pearson, 2008 [21]	Why Children Die: A Pilot Study.	Confidential Enquiry into Maternal and Child Health.	Domain 1
Kool, 2015 [22]	Febrile children at a general practice out-of-hours service.	PhD thesis on the management of fever in children in GP practices.	Domain 1
Morley, 1991 [23]	Field trials of the Baby Check score card in general practice.	Observational study aiming to assess the efficacy of a tool to identify children at risk of severe disease.	Domain 1
Hjortdahl, 1991 [24]	C-Reactive Protein: A New Rapid Assay for Managing Infectious Disease in Primary Health Care.	Diagnostic test accuracy study of CRP.	Domain 2
O’Brien, 2019 [25]	CRP POCT to guide antibiotic prescribing in primary care settings for acute respiratory tract infections.	Health technology assessment of CRP POCT.	Domain 2 and 6
Hopstaken, 2003 [26]	Contributions of symptoms, signs, erythrocyte sedimentation rate, and C-reactive protein to a diagnosis of pneumonia in acute lower respiratory tract infection.	Diagnostic test accuracy study of CRP.	Domain 2
Van Vugt, 2013 [27]	Use of serum C reactive protein and procalcitonin concentrations in addition to symptoms and signs to predict pneumonia in patients presenting to primary care with acute cough: diagnostic study.	Diagnostic test accuracy study of CRP.	Domain 2
Minaard, 2015 [28]	The added diagnostic value of five different C-reactive protein point-of-care test devices in detecting pneumonia in primary care.	Diagnostic test accuracy study of CRP POCT.	Domain 2
Van den Bruel, 2011 [29]	Diagnostic value of laboratory tests in identifying serious infections in febrile children.	Systematic review of the diagnostic test accuracy of various biomarkers including CRP to predict serious bacterial infections.	Domain 2
Kool, 2016 [30]	C-Reactive Protein level as diagnostic marker in young febrile children presenting in a general practice out-of-hours service.	Diagnostic test accuracy study of CRP POCT.	Domain 2
NHG, 2011 [31]	NHG Guidelines for acute cough.	Guidelines from the Dutch college of GPs on cough.	Domain 2
NHG b, 2011 [32]	NHG Guidelines on diverticulitis.	Guidelines from the Dutch college of GPs on diverticulitis.	Domain 2
NHG, 2021 [33]	NHG Guidelines on Chronic Obstructive Pulmonary Disease.	Guidelines from the Dutch college of GPs on COPD.	Domain 2
NICE, 2014 [34]	NICE Clinical guideline on pneumonia in adults: diagnosis and management.	National Institute for Health and Care Excellence’s guidelines for the management of pneumonia.	Domain 2, 3, and 6
Howick, 2014 [13]	Current and future use of point-of-care tests in primary care: an international survey in Australia, Belgium, The Netherlands, the UK and the USA.	Survey about the availability of POCT tests in primary care.	Domain 3
Kip, 2019 [17]	Understanding the adoption and use of point-of-care tests in Dutch general practices using multi-criteria decision analysis.	Case study to guide POC test development and their introduction in clinical practice.	Domain 3
Cals, 2009 [35]	*Effect of point of care testing for C- reactive protein and training in communication skills on antibiotic use in lower respiratory tract infections.*	Randomised trial to assess the efficacy of CRP POCT to reduce antibiotic prescription in primary care.	Domain 3
Cals, 2010 [36]	Point-of-care C-reactive protein testing and antibiotic prescribing for respiratory tract infections.	Randomised trial to assess the efficacy of CRP POCT to reduce antibiotic prescription in primary care.	Domain 3
Little, 2013 [37]	Effects of internet-based training on antibiotic prescribing rates for acute respiratory-tract infections: a multinational, cluster, randomised, factorial, controlled trial.	Randomised trial to assess the efficacy of CRP POCT to reduce antibiotic prescription in primary care.	Domain 3
Weesie, 2017 [38]	CRP point of care testing and prescribing antibiotics at the GP post.	Before-and-after evaluation of the use of CRP POCT in primary care.	Domain 3
Little, 2019 [39]	Antibiotic Prescribing for Acute Respiratory Tract Infections 12 Months After Communication and CRP Training: A Randomized Trial.	Long term analysis of a randomised trial on the effectiveness of CRP POCT to reduce antibiotic prescription.	Domain 3
Cals, 2011 [40]	C-reactive protein point of care testing and physician communication skills training for lower respiratory tract infections in general practice: economic evaluation of a cluster randomized trial**.**	Cost-effectiveness analysis of C-Reactive Protein POCT to reduce antibiotic prescribing in primary care.	Domain 3
Holmes, 2018 [41]	Cost-Effectiveness Analysis of the Use of Point-of-Care C-Reactive Protein Testing to Reduce Antibiotic Prescribing in Primary Care.	Cost-effectiveness analysis of C-Reactive Protein POCT to reduce antibiotic prescribing in primary care.	Domain 3
Kroneman, 2016 [42]	The Netherlands Health system review.	In-depth review of the Dutch health system.	Domain 5 and 6
Cylus, 2015 [43]	United Kingdom Health system review	In-depth review of the British health system.	Domain 5 and 6
UK Government, 2014 [44]	International comparisons of selected service lines in seven health systems.	Case study describing GP posts in the Netherlands.	Domain 5 and 6
Mossialos, 2017 [45]	International profiles of healthcare systems, 2016.	In-depth review of the Dutch health system.	Domain 5 and 6
Mguire, 2011 [46]	Which urgent care services do febrile children use and why?	Observational study aiming to explore how parents navigate urgent and emergency care services when their child < 5 years old has a feverish illness	Domain 5 and 6
Wolfe, 2016 [47]	Child Health Systems in the United Kingdom (England)	In-depth review of child health services in England.	Domain 5 and 6
Bentum, 2018 [48]	Determining factors that influence purchasing laboratory services in primary care.	MSc dissertation	Domain 5
NVKC, 2015 [49]	Guidelines: Point of care testing (POCT) in general practice.	Dutch College of GPs and the Dutch Association for Clinical Chemistry and Laboratory Medicine guidelines for the use of POCT tests in primary care.	Domain 5
NHS, 2020 [50]	Diagnostics recovery and renewal.	Independent review of the diagnostic services for NHS England.	Domain 5 and 7
Wammes, 2020 [51]	International Health Care System Profiles: Netherlands	In-depth review of the Dutch health system.	Domain 5
Nuffield trust, 2014 [52]	The NHS payment system: evolving policy and emerging evidence.	Independent review of the NHS payment system.	Domain 5
NHS England, 2020 [53]	Delegated commissioning of primary medical services.	NHS England website.	Domain 5
UKADC, 2018 [54]	CRP & POC Accelerated Learning Workshop 2018	Workshop organised by NHS England to explore facilitators and barriers to the implementation of CRP POCT in the NHS	Domain 5
Dutch government, 2015 [55]	Tackling antimicrobial resistance, the Dutch one health approach.	Summary of the Dutch antibiotic resistance policy.	Domain 6
ECDC, 2019 [56]	Antimicrobial consumption in the EU/EEA	Report on trends in antimicrobial consumption for systemic use in the community (primary care sector) in Netherlands, United Kingdom from 1997 to 2019	Domain 6
UK government, 2013 [57]	UK 5-year antimicrobial resistance strategy 2013 to 2018.	British antimicrobial resistance plan.	Domain 6
Anyanwu, 2019 [58]	Conceptualising the Integration of Strategies by Clinical Commissioning Groups in England towards the Antibiotic Prescribing Targets for the Quality Premium Financial Incentive Scheme: A Short Report	Qualitative study reporting antimicrobial stewardship measures used by CCGs.	Domain 6
van der Linden, 2001 [59]	Integration of care in The Netherlands: the development of transmural care since 1994.	National survey to determine the success of the bottom-up policy and the extent of the development of transmural care.	Domain 6
Maile, 2022 [60]	Back to the future? Lessons from the history of integrated child health services in England.	Review of the history of integration in the English National Health Service.	Domain 6
European Commission, 2021 [61]	CE marking.	Information on the European Union’s single market standards.	Domain 6
UK Accreditation Standards, 2022 [62]	Point of care testing accreditation.	UK standard for POC accreditation	Domain 6
NZA, 2011 [63]	Decisions of the Board of Directors October 11, 2011.	Official decision by the Dutch Health Authority to include CRP POCT in the list of reimbursable consumables.	
Thomson, 2020 [64]	Private Health Insurance history politic and performance	In-depth review of the private health insurance schemes	Domain 6
European Commission, 2016 [65]	The Netherlands Health Care & Long-Term Care Systems.	In-depth review of the Dutch health systems by the European Commission.	Domain 6
Borisenko, 2018 [66]	Innovative payment schemes for medical technologies and in- vitro diagnostic tests in Europe.	Report by the European invitro diagnostic industry association about reimbursement schemes.	Domain 6
Derksen, 2011 [67]	Medical tests (assessment of established medical science and medical practice).	Processes for the evaluation of diagnostics by the Zoorg Institute Netherlands.	Domain 6
Thomson, 2009 [68]	Financing healthcare in the European Union.	In-depth review of financing mechanisms for healthcare	Domain 6
Anderson, 2021 [69]	Re-laying the foundations for an equitable and efficient health and care service after COVID-19	LSE-Lancet Commission on the future of the NHS.	Domain 6
OECD, 2020 [18]	Health spending*,* 2020*.*	Report on health spending in countries member of the Organisation for Economic Cooperation and Development.	Domain 6
Steel, 2018 [70]	Changes in health in the countries of the UK and 150 English Local Authority areas 1990–2016: a systematic analysis for the Global Burden of Disease Study 2016.	Systematic analysis of the burden of disease in the UK.	Domain 6
ROS Robuust 2021 [71]	ROS Robuust.	Website of regional support organization	Domain 6
AHSN, 2021 [72]	Academic Health Science Network.	Website of national support organization	Domain 6
Van den Bruel, 2016 [73]	C-reactive protein point-of-care testing in acutely ill children: a mixed methods study in primary care.	Randomised clinical trial in England.	Domain 6
UKADC, 2021 [74]	United Kingdom Antimicrobial Diagnostics Collaboration	Website of public institution supporting the implementation of diagnostics.	Domain 6
Johnson, 2018 [75]	Funding and policy incentives to encourage implementation of point-of-care C-reactive protein testing for lower respiratory tract infection in NHS primary care: a mixed-methods evaluation.	Implementation research study about the the introduction of a reimbursement scheme for the adoption of CRP POC tests.	Domain 6
Eley, 2020 [76]	Effects of primary care C-reactive protein point-of-care testing on antibiotic prescribing by general practice staff: pragmatic randomised controlled trial, England, 2016 and 2017.	Randomised clinical trial in England.	Domain 6
Wakeman, 2018 [77]	Point-of-care C-reactive protein testing in community pharmacy to deliver appropriate interventions in respiratory tract infections (RTIs)	Study investigating the feasibility of a rural community pharmacy offering this service and delivering the most appropriate intervention in RTIs.	Domain7
Zorginstituut, 2019 [78]	Report initial meeting Sensible Care Lower respiratory tract infection and pneumonia.	Minutes of the meeting of the Zoorg Institute working group assessing the quality of primary care	Domain 7
Review on Antimicrobial Resistance, 2015 [79]	Rapid diagnostics: stopping unnecessary use of antibiotics. The review on antimicrobial resistance.	Independent review on antimicrobial resistance commissioned by the UK government.	Domain 7

**Table 2:** Characteristics of stakeholders

Stakeholders	Netherlands	England
In vitro diagnostics industry representatives	1 (F)	1 (M)
Health insurance company representative	1 (M)	–
Clinical commissioning group member	–	1 (M)
Clinical guidelines development group member	1 (M)	1 (M)
Member of NHS quality improvement programme (NHS Improvement)	–	1 (F)
CRP POCT tests implementors in primary care	1 (M, head of hospital laboratory)	1 (F, Nurse Practitioner)
General practitioners		
Consultants	4 (2F)	5 (1F)
Trainees	2 (1F)	1 (F)
**Total**	**10**	**11**

*F* Female, *M* Male

The analysis identified similarities and differences in the seven NASSS domains between the two countries (Table 3) and are presented narratively below. In the narrative we intertwined data from the documents and the interviews pertaining to each domain of the NASS framework to synthesise the findings.

**Table 3:**
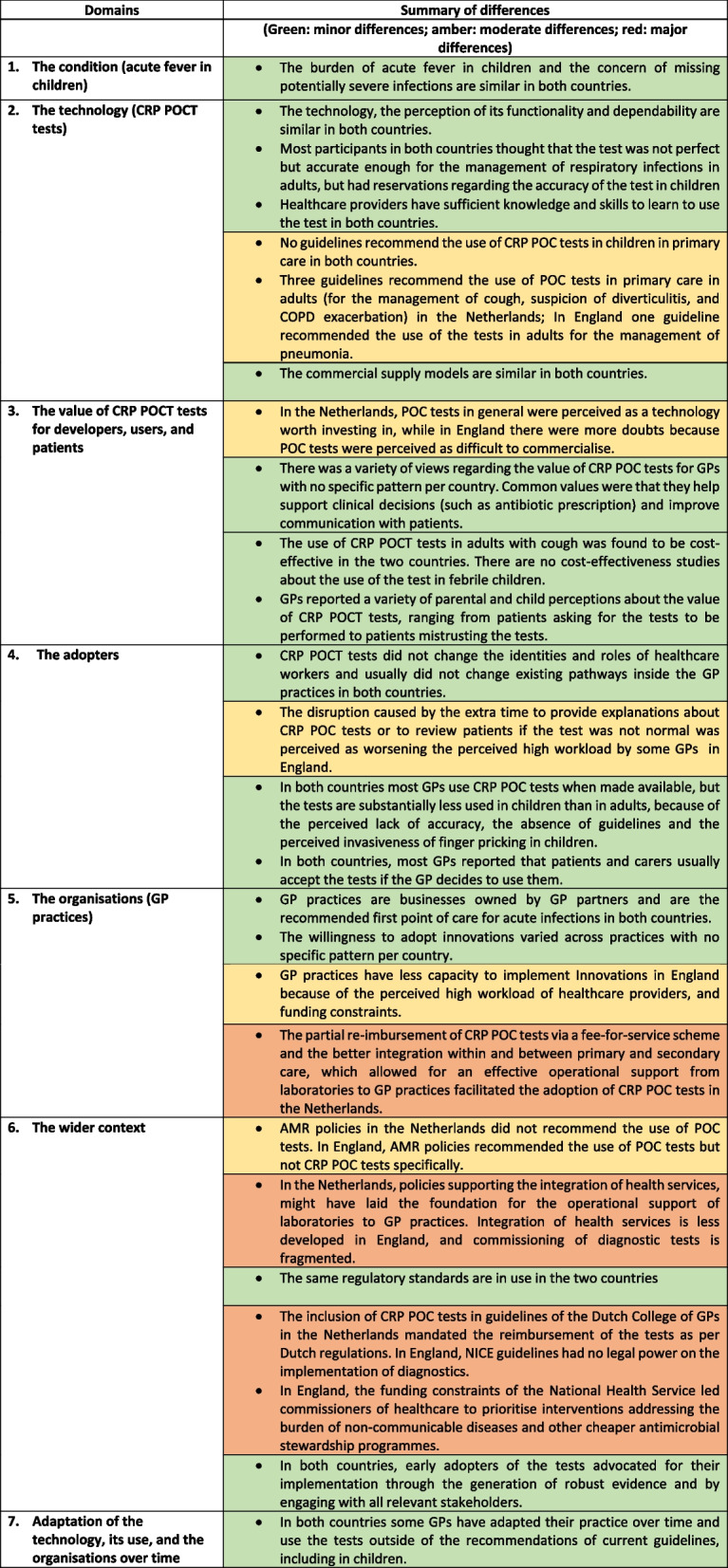
Summary of differences in the NASSS domains that explain the difference in adoption of CRP POCT between the Netherlands and England

*CRP* C-reactive protein, *POCT* Point-of-care test, *GPs* General practitioners, *COPD* Chronic obstructive pulmonary disease, *NICE* National Institute for Health and Care Excellence

### The condition

The condition is acute fever in children. There are few differences between the two countries regarding the burden of the condition in primary care. Fever in children usually indicates the occurence of an infection and is a common cause of consultation: in the Netherlands it is estimated that around 31% of children consulting in primary care are presented by their carers because of fever [19], while in England it is 20–39% [1]. Infections are one of the leading causes of death in children, with 23 and 20% of child deaths caused by infections in the Netherlands [20] and in England [21], respectively. However, < 5 and 3.5% of children presenting to primary care services are estimated to have severe infections in the Netherlands [22] and in England [23], respectively.

In both countries, most of the interviewed GPs expressed concern about missing severe infections in children:

*“We send too many children to a paediatrician because we are just afraid to miss one case of severe infection” (GP3-Netherlands).*

*“[we are] … very, very careful (with children)” (GP4-England).*

### The technology

#### Material features and type of data generated

CRP POC tests were initially developed in Norway [24] and Finland [25]. They are available as a quantitative or a semi-quantitative test. We only considered the quantitative devices, as these are the devices implemented in the two countries and that are the object of the documents included in this study. There are currently twelve quantitative CRP tests available [25]. They are cartridge based tests where a droplet of blood, usually obtained by finger prick, is placed in a cartridge that is then inserted into a small mains-powered analyser. The results are usually available within 5 minutes and displayed as a digital read out of blood CRP concentration in mg/L.

The accuracy of CRP POC tests varies according to the condition for which the test is used [25]; Several studies found that the accuracy of CRP in the management of low respiratory tract infection in adults is good, although not perfect [26–28]. With regards the accuracy in febrile children, CRP is one of the best biomarkers to identify severe infections in children [29]. However, the accuracy of rapid CRP POC tests in febrile children in primary care settings is still debated [30]. In this study, GPs reported using the tests mainly for managing adults with cough and were more uncertain about the accuracy of the test in children:

*“I am not quite so convinced that a normal CRP would mean they actually are quite well, they don’t have a bacterial infection” (GP3-England).*

#### Knowledge and support to use the tests

Most participants in both countries thought that the tests were quick and easy to use:

“much easier (than venous sampling), it’s quicker, it’s simple, it’s clean” (GP2-Netherlands).

“to get the test back in four minutes is fantastic” (GP1-England).

Ideally, the use and interpretation of results should be informed by clinical guidelines. Guidelines from the Dutch Royal College of GPs recommend the use of CRP POC tests for the management of adults with cough [31], suspected diverticulitis [32], and exacerbation of COPD [33]. The tests are only recommended in patients with diagnostic uncertainty to help in deciding whether antibiotics should be prescribed. In England, one guidelines from the National Institute for Health and Care Excellence (NICE) recommends the use of CRP POC tests in adults with suspected pneumonia which is similar to the Dutch guidelines [34]. In both countries, there are no guidelines that recommend the use of CRP POC tests in primary care in children.

Support of actual implementation of the tests is covered in The organisations.

#### Adaptation of the technology and supply model

The implementation of POC tests is complex and involves several actors and processes (see The adopters (healthcare providers and patients), The organisations, and The wider system) but from a technological point of view, CRP POC tests are relatively straightforward devices that do not need to be specifically adapted prior to their implementation in any healthcare facility.

Some of the manufacturers of the tests are large multinational companies [25] which supply both the Netherlands and England. This means that the tests can be purchased and obtained in the two countries in similar ways.

### The value proposition

In the Netherlands, the availability of CRP POC tests in GP practices is high with estimates of between 48% [13] and 80% [17] in 2014–2015. By contrast, the availability of CRP POC tests in GP practices in England is much lower but data are scanty. One survey conducted in 2014 reported availability to be 15% in 2014 [13].

The potential “value” of the test depends on the perspective i.e., whether it is the perspective of industry (“supply-side), or individual GPs or health care commissioners (“demand-side”).

#### Supply-side value

From the perspective of the in-vitro diagnostic industry, there is revenue potential in Netherlands:

*“Everybody says that they expect that it is becoming more and more, popular, and that the growth in diagnostic industry will be in point of care and not in lab tests.” (*In-vitro *diagnostics industry representative-Netherlands).*

Whereas with regards the market in England, POC tests in general were seen as “a tough sell still” and whether there was demand for it in primary care was perceived “debatable” (In-vitro diagnostics industry representative-England).

#### Demand-side value

There is strong evidence that the introduction of CRP POC tests can reduce antibiotic use. This includes two randomized controlled trials (RCT) conducted in the Netherlands [35, 36] and one conducted in five countries (including the Netherlands and England) [37]. A before-and-after evaluation based on routine data collected from GP practices also found that the use of antibiotics decreased after the introduction of the tests [38]. However a long-term impact analysis of the multi-country RCT showed that the effect of the intervention did not last at 12 months of follow up [39]. With regards cost-effectiveness, studies also suggest that the use of CRP POC tests is cost-effective in the pathway of care for adults with pneumonia in both countries [34, 40, 41]. Cost-effectiveness of using the tests in children in primary care has not yet been examined.

All the GPs in the Netherlands said that they found the tests very useful and none of them knew of other GPs who did not use them. Some said that although initially they were not particularly interested in the tests, this changed rapidly:

*“We had it and, as soon as we use it, we didn’t want to give it back.” (GP2-Netherlands).*

As with their counterparts in Netherlands, all the GPs in England who had experience of using the tests in pilot studies said that they found them useful, but recognized that their views were not always shared by others:

*“The other doctors are not at all convinced and so I think we never really got into a culture of using them a lot except for me” (GP1-England).*

In both countries CRP POC tests were commonly said to help the decision to prescribe antibiotics, resulting in a perceived reduction in antibiotic use athough one interviewee from the Netherlands expressed that this effect might not last, based on the long-term impact analysis of the multi-country trial referred to above [39]. 

CRP POC tests were also perceived as helping to avoid sending patients to distant laboratories, a major difficulty expressed by most respondents from both countries.^.^ Another advantage of CRP POC tests for GPs in both countries was that it supported decisions and improved communication with patients, including with children:

*“If I can’t convince them (that antibiotics are not needed) myself I do it with the test” (GP2-Netherlands).*

*“They (children) loved having the test done and they wanted to know about it, and it was a chance to say most infections are viral and this shows you don’t need antibiotics” (GP1-England).*

Most GPs in both countries thought that CRP POC tests were not useful to inform decisions as to whether to refer a patient to hospital or not. However, a few GPs disagredd and did think they helped with this decision:

*“If that (the need to refer) is really the case then you should already be able to see if the patient is really ill, and I don’t think that the CRP, should make any difference in that” (GP3-Netherlands).*

*“It’s more than just “I’ll prescribe some antibiotics”, it also helps to decide whether someone should be admitted to hospital” (GP3- England).*

There were mixed views in both countries about the utility of using the tests in children, some expressing uncertainty about their added value, whilst others being more positive:

*“There are lots and lots of kids we see with high fever, no diagnostic, no pointers to anything serious – it’s a very common situation, and in that situation point-of-care testing would be very helpful” (GP1-England).*

None of the interviewees were aware of whether the tests were considered cost-effective despite there being several studies as mentioned above.

Patients were not interviewed as part of this study. However, from the perspective of the GPs, some of them in the Netherlands reported that some patients wanted the tests to be used, particularly when they disagreed with the GP’s decision or when they sought reassurance. In England, GPs reported mixed reactions with some patients liking the tests as it suggested to them that they were being taken seriously, whilst others being more mistrustful:

*“It’s almost like they go to hospital, and they turn up in Accident & Emergency” (GP2-England)*

“*Some patients go:* “I don’t trust your machine doctor*”” (GP3-England).*

For GPs from practices where CRP POC tests were unavailable in England, there was a perception that if they were made available there would be demand for their use, which may not necessarily be a good thing:

*“It would increase demand and then you risk that any child with an upper respiratory tract infection will cost you four pounds” (GP4-England).*

### The adopters (healthcare providers and patients)

#### Changes in staff roles, practices, and identities

In this study the staff are GPs and practice nurses or assistants. In both countries, the implementation of CRP POC tests was not perceived to have changed their identities or practices, with the GPs being responsible for seeing the patient and ordering the test, and the test then usually being performed by nurses or assistants. GPs saw the patient a second time only if the results were out of the normal range. This care pathway was like other pathways including the use of urine dipsticks or electrocardiograms and was perceived as “the normal work” (GP5-Netherlands). Having to see the patient a second time was perceived by all GPs in the Netherlands as acceptable. This could have contributed to the adoption of the tests by all interviewed GPs in the Netherlands.

By contrast, in England, GPs had more mixed views. Some thought the disruption was acceptable, while others thought using CRP POC tests extended the consultation time because they had to provide more information to patients and any increase in consultation time, even marginal, and/or seeing the patient again was perceived as difficult:

*“We had to tell them what is CRP, what does it mean, why does it mean that they don’t need antibiotics, and what is the difference between a virus and a bacteria. It actually added more layers, layers of communication” (GP3-England).*

*“(Doctors and nurses) … ..don’t want to be messing around with three minutes, they are busy, very, very busy” (POC test implementor-England).*

With regard to using the tests in children, in the Netherlands some GPs never used them in this age group while some did, but much less frequently than in adults. In England only one GP used CRP POC tests in children but also less frequently than in adults. In both countries, the reasons given included concerns about their accuracy and the absence of any reference to their use in children in guidelines. Some GPs in England also found that finger pricking in children was invasive, and causing pain was perceived as undesirable.

#### Percieved acceptability by patients

All GPs in the two countries expected and reported that patients, including children, accepted the tests, if the GP decided to use it.

### The organisations

**Fig. 2:**
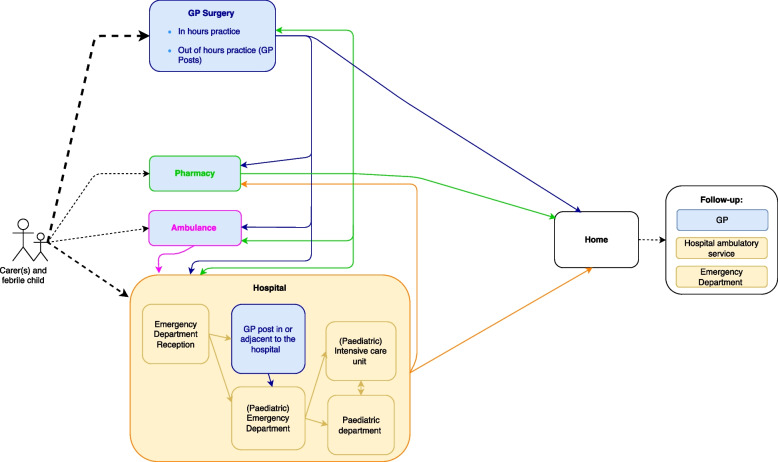
General Practitioner practices in the care pathways for febrile children in the Netherlands. GP: General practitioner

**Fig. 3:**
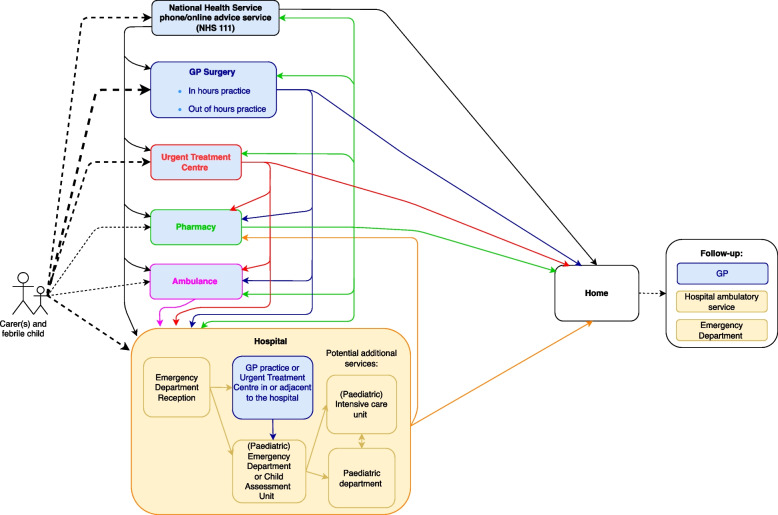
General Practitioner practices in the care pathways for febrile children in the England. GP: General practitioner.

In this study “organisations “refers to GP practices. In both countries GP practices are businesses that are run by GPs [42, 43]. Their role in the care pathways for febrile children in the two countries is similar: they are the recommended first point of care and act as gatekeepers of other health services. However, some parents present directly to emergency departments, call an ambulance, or ask for advice from a pharmacist [19, 42, 44, 45]. There are a few more options in England, such as telephone and online triage services and urgent treatment centres that can be accessed without appointments (Figs. 2 and 3) [42, 46, 47].

#### Capacity to innovate

Most participants in both countries reported that the willingness and leadership to implement innovations varied across workplaces; some practices being keen to take-up innovations while others were perceived as being conservative and reluctant to try new approaches. As independent businesses, GP practices are free to decide whether they want to adopt diagnostics in both countries. However, the GPs interviewed in England expressed that their capacity to adopt CRP POC tests was limited by their perceived heavy workloads, which made training and integrating new ways of working difficult, and because of the lack of financial support (see below).

#### Readiness for the implementation of CRP POC tests

In the Netherlands, hospital laboratories and primary care laboratories play an important role in implementing the tests in GP practices and in ensuring the tests are used in line with regulatory standards (see The wider system) [48], as per the recommendations of the Dutch College of General Practitioners (NHG), the Dutch Association for Clinical Chemistry and Laboratory Medicine (NVKC), the Dutch Society for Medical Microbiology (NVMM), and the Laboratories Physicians Collaboration Netherlands (SAN) [49]. The existing operational support of laboratories to GP practices is likely to contribute to the readiness of the latter to implement CRP POC tests. In this study two of the three GP practices reported already having contracts with hospital laboratories that supported the implementation of other diagnostics prior to the introduction of CRP POC tests.

In England, there are very few primary care laboratories [50] and the capacity of hospital laboratories to support the implementation of diagnostic tests in GP practices is limited. Several participants mentioned that GP practices were not ready to implement CRP POC test at scale and in a sustainable way, and that this was in part because of the lack of support from hospital laboratories:

*“They are busy enough inside (the hospital), if they want to come to see five practices within 20 miles, one person in a car driving out, whereas they got hundreds of machines in the lab, so they don’t want to spend time travelling” (POC tests implementor-England).*

Integration of health services (or the lack of) appears to be an important factor in the adoption of CPR POC tests in GP practices. This is explored more in detail in The wider system.

#### Funding decision

In the Netherlands, GP practices are financed by health insurance companies. These companies are mainly funded by the premiums received from the insured population (i.e., all residents, as being covered by health insurance is mandatory in the Netherlands) [42]. The funding that GP practices receive consists of a combination of capitation, fee-for-consultations, bundled payments for integrated multidisciplinary care for chronic conditions, and pay-for-performance focused on accessibility and referral patterns [51]. In addition, few fee-for-service schemes exist, including a scheme that partially reimburses the use of CRP POC tests. As mentioned earlier, only the consumables to operate each test are reimbursed. The analyser must be purchased without reimbursement by the primary care or hospital laboratory that supports and implements the test in the GP practices as a capital investment.

In England, GP practices are funded by clinical commissioning groups (CCGs). CCGs are groups of general practices which commission health services for the population of their area. CCGs receive their funding from the national health service (NHS England) and allocate funds to each GP practice. The amount of funding received by GP practice is made up of a combination of capitation, pay-for-performance, fee-for-service, and additional funding for the maintenance of premises and seniority primes [43, 52, 53]. There are no pay-for -performance or fee-for-service schemes that fund the use of CRP POC tests, which means that GP practices need to pay for the full cost of using the tests from their budget.

#### Work needed to implement change

The implementation of CRP POC tests was seen as easy and straightforward by GPs in the Netherlands and, for some GPs, the absence of CRP POC tests was actually more problematic than its implementation, suggesting that test is highly normalised:

*“We only had to make room for the machine. And our assistants got some guidance of how they had to do the test” (GP5-Netherlands).*

*“Sometimes it’s broken, then we don’t have one … That’s the problem. That’s basically it.” (GP-5 Netherlands).*

By contrast, in England, implementation was perceived as difficult. All the interviewed GPs mention that their practice would not want to pay for the tests from their budget. They would need to obtain funding form charities or convince the CCGs to allocate additional funding and then set up an agreement with a local hospital or directly with a diagnostic test company for technical support. All of this was perceived as very difficult:

*“Why should they (the CCG) invest this amount of money in a CRP project. So, I must say the fight to get mine was quite intense and I had to be very persistent” (POC tests implementor-England).*

This was mainly caused by the funding constraints that CCG face (see The wider system). Because of this, GP practices had to conduct pilot studies to convince CCGs of the clinical and cost-effectiveness of CRP POC tests use in the local care pathways. This led to the proliferation of pilot studies: In 2017, there were 34 pilot studies across the UK involving the use of CRP POC tests in primary care [54].

### The wider system

#### Policy context

Policies pertaining to antimicrobial resistance (AMR) were examined because the use of diagnostic tests has been advocated as a means to reduce antibiotic use. The Dutch AMR policy recommends the use of new diagnostics to contain AMR but does not specifically mention POC tests [55]. Despite this, the implementation of CRP POC tests is one of the main antimicrobial stewardship measures in primary care [38]. The already low rate of antibiotic prescription in primary care decreased by 14% since 2011 [56], and the use of CRP POC tests has probably contributed to this decrease. In England, the UK AMR policy supports the use of POC tests generally but do not specifically mention CRP [57]. CCGs usually choose other antibiotic stewardship measures that have no or little additional costs over diagnostics (such as setting antibiotic prescription targets, or benchmarking the use of antibiotics across GP practices and CCGs). The prescription of antibiotics decreased by 16% in primary care in the UK between 2014 and 2019 [56], and some participants in England stressed that this was achieved because of these alternative antimicrobial stewardship (AMS) interventions [58].

We also examined policies pertaining to the integration of health services, because the support of primary care and hospital laboratories to GP practices for the implementation of CRP POC tests was an important factor in the Netherlands. The concept of “transmural care” i.e., the integration within primary care and between primary and secondary care has been promoted to improve the quality of healthcare since the 1990s in the Netherlands. Since then, transmural care has become a common aspect of the organisation of health services, even though there is still room for improvement [59]. By contrast, integration within and between levels of healthcare is still in development in England, despite several policies promoting it [60].

#### Regulatory context

All available CRP POC tests are CE marked in accordance with the European Union IVD Directive (98/79/EC) [25]. CE marking is a process through which the manufacturer self-declares that the device conforms with EU regulatory standards [61]. This allows manufacturers to commercialise their products legally in the EU, including the Netherlands and England (until December 2020 for the latter).

The relevant International Standards Organization (ISO) standards are followed in both countries. These are ISO 15189 for the general laboratory activities of laboratories supporting GP practices and ISO 22870 for the specific use of POC tests [49, 62].

#### Role of professional bodies

In the Netherlands, the Dutch Royal College of GPs played a key role in establishing the role of CRP POC tests in primary care. The use of these tests was recommended in clinical guidelines developed by the Dutch Royal College of GPs since 2011. This led the Dutch Healthcare Authority (NZA), an independent organisation that sets tariffs for the reimbursement of health services, to include CRP POC tests (but not the analyser) in the list of medical devices that can be reimbursed to primary care services [63]. A tariff listed by the NZA mandates the reimbursement of the tests by health insurance companies:

*“And we have to pay because by law, we have to ensure that they can get all the necessary care they need” (health insurance company representative-Netherlands).*

This is because health insurers must reimburse health interventions that are included in a package called the Basic Package of Care [42, 64]. The government decides the content of the Basic Package of Care [42, 65] usually following the recommendations of the Zorg Instituut Nederland, another independent body in charge of health technology assessments (HTAs) [42, 45, 64, 66]. In theory, healthcare, including diagnostics, must be “normally provided by healthcare workers” and supported by “evidence of clinical and cost effectiveness” to be supported by the Zorg Instituut [65, 67]. In practice, healthcare that is recommended in clinical guidelines is considered “normal” care and is almost automatically included in the Basic Package of Care; a reimbursement tariff is then set by the NZA. The Zorg Institute does not necessarily carry out prior HTAs, particularly if the innovation is not substantially expensive, which is the case of CRP POC tests [42, 65, 67].

In England, although the 2014 NICE guideline on pneumonia recommended the use of CRP POC tests in the management of adults with suspected pneumonia [34], these had limited impact in terms of their implementation in GP practices. The guidelines were produced with input from key stakeholders including the Royal College of GPs, however, NICE guidelines are only advisory and do not mandate the funding decisions of CCGs.

#### Financing issues

Both the UK and the Netherlands spend about 10% of GDP in healthcare [18]. However, health expenditure per capita in the UK is 16% lower than in the Netherlands, given than GDP per capita is lower in the UK. Containment of healthcare costs is a common issue across European countries but has been particularly important in the UK since 2010 [68, 69] As a result, funding available to CCGs is relatively limited, and interview participants perceived that CCGs were very constrained financially and had to make difficult decisions about what type of healthcare to prioritise. This was perceived by some participants to lead to more precedence to the treatment of adult non-communicable diseases, because of their greater contribution to the country's burden of diseases [70].

#### Interorganisational networking

In both countries, there are regional support structures which role is to disseminate healthcare innovations, such as for example ROS Robust [71] in the Netherlands and the Academic Health Sciences Network (AHSNs) in England [72]. Additionally, some participants in the Netherlands mentioned the important role that early Dutch adopters played in generating local clinical, cost-effectiveness, and broader evidence about the use of CRP POC tests. They proactively disseminated the evidence and engage with all actors involved in the key decisions and processes that lead to the adoption of diagnostics in primary care. There are also “champions” in England who promoted and continue to promote the implementation of the tests [37, 73–76]. It is difficult to estimate whether the work and intensity of efforts of these early adopters was greater or different across the two countries. However, the overall context described in detail across this paper was, and still is, more favourable and more receptive to the engagement of these actors in the Netherlands.

### Adaptation of the technology, its use, and the organisations over time

#### Scope of adaptation over time

CRP POC tests devices cannot be physically changed or adapted. However, a few GPs in both countries reported that their use had extended beyond the conditions which are the scope of current guidelines (cough, diverticulitis or COPD in the Netherlands; pneumonia in England). In England, a few participants mentioned that this had a negative impact on the perceived value of the tests by CCGs. There were also explorations in England to shift the use of the test to pharmacies [77], yet this has not so far led to its implementation in those settings.

#### Organisational resilience

The concept of “diagnostic stewardship” with regards CRP POC tests has been gaining attention in the Netherlands. In 2018 the Zorginstituut launched a consultation of experts to improve the management of respiratory infections in primary care. One area of concern was the use of CRP POC tests in children, as this is not recommended in current guidelines and was reported to the Zorginstituut by primary care experts informing the consultation [78]. The consultation will provide its recommendations in 2023.

In England, several recent reviews commissioned by the department of health on AMR and on improving the diagnostic capacity of the NHS have advocated for more adoption of POC tests [50, 79]. CRP POC tests are cited as an example of POC tests which could contribute to improving antibiotics use, which suggest that these tests have not completely been ruled out, despite the current barriers to their implementation. Many participants felt that the only way to implement CRP POC tests at scale in England would be that it is mandated by NHS England with a specific funding scheme:

*“It’s only, it’s only when things are mandated that things will get, done, really done” (Clinical commissioning group member-England).*

## Discussion

### Summary of principal findings

A more favourable and receptive macro level environment combined with the endeavour and engagement of early adopters led to the successful adoption of the tests in the Netherlands. In the two countries, early adopters of the tests advocated for their implementation through the generation of robust evidence and by engaging with all relevant stakeholders. Their work was essential in creating awareness about the tests and the evidence supporting their use among the actors involved in the adoption of diagnostics in health services. This led to the inclusion of CRP POC tests in national clinical guidelines in both countries. In the Netherlands, this resulted in the cost of the tests being partially reimbursed under a fee-for-service reimbursement mechanism. Moreover, the prevailing integration of health services enabled operational support from primary care and hospital laboratories to GP practices for the implementation of the tests. In England, the guidelines were only advisory and did not result in any mandates in relation to the use of or the reimbursement for CRP POC tests. Moreover, funding constraints and the resulting prioritization of less expensive antimicrobial stewardship interventions, the lack of integration across health services, the lack of operational support to GP practices, and the resulting perception that the introduction of CRP POCs would be a source of additional expenses and workload have all contributed to CRP POC tests not being adopted.

With regards the use of CRP POC tests in children with fever, this was often seen to be a by-product of the test being made available for adult patients. In both countries, the tests are rarely used in children. This is mainly because of concerns about the accuracy of the tests in children, the lack of guidelines specific for this age-group, and the perceived invasiveness of finger pricking in children.

### Comparison with other literature

Other studies have investigated some of the different facilitators and barriers to the availability and use of POC tests in primary care presented in this paper.

We found that CRP POC tests were valuable for GPs for various reasons, with no distinctive pattern per country. Another study exploring the value of POC tests for GPs across European countries found that there was a variety of positive and negative views, and that these were shared across countries [80]. Better targeting of antibiotic use and supporting decisions and communication with patients were among the most cited values, which is in keeping with our findings.

We found that the interplay between early adopters and the overall context contributed to the adoption of the tests. In a study exploring the facilitators and barriers to the adoption of CRP POC tests in Northern European countries, Huddy and colleagues found that the work of early adopters was essential in facilitating the adoption of the tests because the early adopters acted in a favourable environment that encouraged POC technology and the reduction of antibiotic prescription. This in turn allowed the development of reimbursement schemes that supported large-scale adoption [14] and is line with our study. A recent health technology assessment of CRP POC tests found that of 11 European countries that implemented CRP POC tests in primary care, a reimbursement scheme was available in seven countries (not data was available for the remaining four countries) [25], which suggest that reimbursement schemes contribute to the adoption of the test, which is again in keeping with our findings.

Funding constraints in England was one of the major barriers to the implementation of CRP POC tests in our study. An independent review about the introduction of innovations in the NHS found that funding restrictions was limiting the adoption of innovations [81]. The most recent UK National Action Plan against AMR suggests that this was particularly true for diagnostics and that “if a new promising diagnostic came out tomorrow, the NHS is not equipped to get it into front-line use quickly” [82].

Our study found that the tests were reportedly used less in children than in adults. Schot and colleagues in a qualitative study with GPs in the Netherlands also found that GPs use substantially less CRP POC tests in children because of concerns regarding the lack of accuracy and the invasiveness of the tests [83].

### Strengths and limitations

To the best of our knowledge, this is the first study to use the NASSS framework to compare the adoption of a healthcare innovation in two countries. Using the framework allowed us to conduct an in-depth, comprehensive, and consistent comparative health systems analysis. We conducted a document analysis in combination with interviews of a wide range of stakeholders in the two countries which allowed us to triangulate most of the findings presented in this article. Moreover, most studies on the adoption of POC tests focus on the adoption of the tests in adult patients; this is one of the few studies exploring the adoption of POC tests for the management of childhood infections. Our findings should be interpreted in light of some limitations, such as the small sample size for the different subgroups of stakeholders, the fact that we couldn’t interview children and their carers, and the possibility that the background and experience of using POC tests by some of the authors may have created bias in the interpretation of data, despite the best attempts to limit this. Moreover, it is important to bear in mind that the qualitative data obtained from the interviews are the perceptions of participants and are not necessarily factual data.

### Implications for organisations implementing POCT tests and future research

This study shows that an in-depth analysis is needed to understand the reasons for the variability in the adoption of diagnostic tests in different countries. The NASSS framework is very useful in this regard.

There is evidence that the use of CRP POC tests can reduce antibiotic use in primary care. As noted earlier, the NHS in England achieved a 16% reduction in antibiotic prescriptions through alternative antimicrobial stewardship measures. This is encouraging, but this rate might be reduced even further if those measures are complemented by the implementation of technologies such as CRP POC tests. However, organizations considering the implementation of POC tests in primary care should carefully consider how the implementation of the tests realistically fits into the wider national context.

Most participants questioned the accuracy and effectiveness of CRP POC tests for the management of febrile children in primary care. Additional research is needed to address these concerns and it may well be that newer and better tests could be transformative. Additional comparative analyses in other settings (i.e., hospitals) and countries and with other POC tests would also be useful to provide additional insights for the implementation of current and future POC tests.

## Conclusion

A more favourable and receptive macro level environment including the influence of clinical guidelines, the funding environment, and the operational support from laboratory services to GP practices, combined with the endeavour and engagement of early adopters have led to the widespeard adoption of CRP POC tests in the Netherlands. In both countries CRP POCT tests, when available, are used much less frequently in children than in adults. This sis mainly because of concerns about their accuracy, the lack of specific guidelines, and the invasiveness of blood testing. These are important factors to consider for any organisation or individuals involved in the development and implementation of POC tests.

## Supplementary Information

**Additional file 1.** 

**Additional file 2.** 

## Data Availability

The datasets generated and/or analysed during the current study will not publicly available but will be available from the corresponding author on reasonable request.
