# Supplementary material for: Adoption of C-reactive protein point-of-care tests for the management of acute childhood infections in primary care in the Netherlands and England: a comparative health systems analysis
Source: BMC Health Serv Res. 2023 Feb 23;23:191. doi: 10.1186/s12913-023-09065-8 (PMC9947887; doi:10.1186/s12913-023-09065-8)
Supplement: Supplementary file 1 — Additional file 1. [file 12913_2023_9065_MOESM1_ESM.docx]

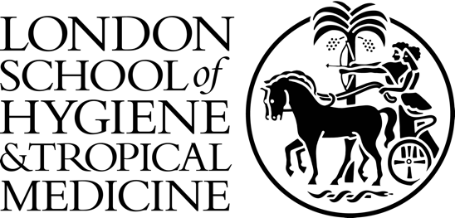
**Topic Guide (GP): Adoption of C-reactive protein rapid tests in primary care**

Participant ID Number: Gender: Male / Female

Country: Date (DD/MM/YY):

**Introduction:**

- Overview and purpose of study
- Who is involved
- Aims of interview and expected duration
- Why participant has been selected
- What will happen to the results of this study
- Questions
- Go through consent form with participant

**Warm-up and general information**:

| For how long have you been working as a GP?  What is your role in the practice?  What is the population affiliated to the practice?   - Adults: - Children:   Which rapid POCTs are available? How many devices are available?  Distance to closest external lab?  What samples are sent there?  Distance to closest hospital? |
| --- |

| **Topics** | **Questions** |
| --- | --- |
| 1. Current practice | A 60-year-old lady that you haven’t met before comes to your practice. She had had fever for 2-3 days (39). She is coughing a bit. She feels tired and has lost appetite.  She hasn’t been in contact with sick people. She has no major health antecedents. She appears a bit tired. Temperature 38.1, HR : 82/ min, RR 21/min, somewhat labored.  Auscultation reveals few rhonchi and few late inspiratory crackles on the right side. The remainder of the lung fields is clear. The rest of the examination is normal   - How would you manage this patient?   - Would you use diagnostic tests?   - Which tests?   - And why?   Now imagine that the patient is a 10-year-old boy. Same story  Same physical examination but with HR 120 and RR 26   - How would you manage this child? - What are challenges, if any when seeing a child with acute fever?   - Would you use diagnostic tests?   - Which tests?   - And why? |
| 1. The technology and its value | - Have you used CRP POCT? - If yes in which circumstances? - What were the advantages/disadvantages of using CRP POCT?   - - For you?     - For the GP practice? - Have you used them in children?   - - If yes, what were the advantages/disadvantages of using CRP POCT in children?     - If no, why? - How did patients/children perceive the use of CRP POCT? - If no, why? - If because tests are not available, let’s imagine the test are made available. - In which circumstances would you use the tests? - What would be the advantages/disadvantages of using CRP POCT?   - - For you?     - For the GP practice? - Would you use them in children?   - - What would be the advantages/disadvantages of using CRP POCT in children? - How would patients/children perceive the use of CRP POCT? |
| 1. The adopters and the impact of CRP POCTs | - What changes, if any, did the use of CRP POCT brought/would bring to:   - the way you work?   - Your role in the practice? - Was the test accepted/ would the test be accepted by patients/parents/children? - Why yes/no? |
| 1. The GP practice | - How innovative in general is your GP practice? - Can you tell me about an innovation that was introduced in your practice? What happened? - How ready was/is your practice for the introduction of CRP POCT? - What problems did you encounter/would you encounter in the implementation of CRP POCT? - Who decided/would decide whether the test should be adopted? - What are the criteria to decide to adopt tests such as CRP POCT? - How are/would be the cost of using the test be covered? - What impact did/would the use of CRP POCTs have on the way your GP practice is organised? - What work was/would be needed in your practice to adopt the test once the decision to implement it is taken? - What were/could be the main challenges in this process? |
| 1. The wider context | - Are you aware of the AMR policy of your country? - What impact does it have on your willingness to implement/use CRP POCT? - What impact does it have on your prescription of antibiotics? - Are there other policies that have an impact on the use of diagnostics/antibiotics? - What role, if any, did your professional association had /could have on the process of implementing tests such as CRP POCT? - How do you get to know about innovations? How is the knowledge about innovations disseminated across GP practices? |
| 1. Adaptation over time | - Has the use of CRP POCT changed since you started using it? Why? - How do you think the use of the tests would evolve if you started using it? - What could change the availability and use of CRP POCTs in the future? |

- Ask participant if he/she has any question
- Ask if there is another relevant person he/she would recommend interviewing
- Ask if there is any document/website he/she would recommend accessing
- Thank participant.
