# Supplementary material for: Adoption of C-reactive protein point-of-care tests for the management of acute childhood infections in primary care in the Netherlands and England: a comparative health systems analysis
Source: BMC Health Serv Res. 2023 Feb 23;23:191. doi: 10.1186/s12913-023-09065-8 (PMC9947887; doi:10.1186/s12913-023-09065-8)
Supplement: Supplementary file 2 — Additional file 2. [file 12913_2023_9065_MOESM2_ESM.docx]

**Supplementary materials 2. Identification of documents for the document review**

## Criteria for considering documents for this review

Documents were included if they pertained to the adoption of diagnostic tests in health services in the Netherlands and England. This included:

- Publications in medical and health systems journals
- Clinical guidelines
- Information for the general public about the use of POC tests in health services
- Information for implementors of diagnostic tests in health services
- Reports and recommendations of organisations involved in the delivery, organisation, funding, regulation, or evaluation of health services, with a focus on diagnostic tests
- Proceedings of conferences

## Search methods for identification of studies

Documents were identified through a multi-pronged approach consisting of:

- Searching databases:
  - Pubmed
  - Google
- Searching websites of organisations involved in the delivery, organisation, funding, regulation, or evaluation of health services in the Netherlands and England:
  - GP practices
  - Clinical commissioning groups
  - Health insurance companies
  - Professional associations of GPs
  - Organisations developing clinical guidelines
  - Local, national, and European health authorities
  - Independent organisations advising these health authorities
  - Agencies in charge of regulating the provision of healthcare
  - Agencies setting tariffs for medical procedures or technology
  - Independent organisations conducting health technology assessments
  - Independent organisations assessing health systems
  - Independent organisations in charge of disseminating innovations in health services
  - the European, Dutch and English in-vitro diagnostics industry
- Searching reference lists of identified documents
- Seeking relevant documents from the 21 interviewees
- Attending relevant meetings and conferences about the implementation of diagnostic tests in health services

The search of data bases and websites were based on the following domains of enquiry:

1. Adoption (i.e., availability and/or use) of diagnostic tests
2. Epidemiology of febrile children
3. Care pathways for febrile children
4. Clinical performance, clinical effectiveness, and cost effectiveness of CRP POC tests
5. Organisation of primary care
6. Funding of diagnostic tests in primary care
7. Regulation of the use of diagnostic tests in primary care
8. Policies pertaining to antimicrobial resistance, integration of health services, and dissemination of technologies in health services

The search was based on a combination of medical subheadings (MeSh), key words, and synonyms for each of the domains of enquiry. The combination of search terms varied and was adapted to ensure it was relevant to the content of each database and websites (i.e., only search terms pertaining to funding of diagnostics were used in websites of organisations involved in the funding of health services).

There were no language restrictions. The search was restricted to 200 to 2019
